# *In vivo* engineering of lymphocytes after systemic exosome-associated AAV delivery

**DOI:** 10.1038/s41598-020-61518-w

**Published:** 2020-03-11

**Authors:** Cort B. Breuer, Killian S. Hanlon, Jeya-shree Natasan, Adrienn Volak, Amine Meliani, Federico Mingozzi, Benjamin P. Kleinstiver, James J. Moon, Casey A. Maguire

**Affiliations:** 1 Center for Immunology and Inflammatory Diseases, Massachusetts General Hospital, Charlestown, MA USA; 2 Department of Neurology, Massachusetts General Hospital, Charlestown, MA USA; 3 Department of Neurobiology, Harvard Medical School, Boston, MA USA; 4 Genethon, INSERM UMR S951, Evry, France; 5 Sorbonne University, INSERM U974, Paris, France; 6 Center for Genomic Medicine, Massachusetts General Hospital, Boston, MA USA; 7 Department of Pathology, Massachusetts General Hospital, Boston, MA USA; 8 Department of Pathology, Harvard Medical School, Boston, USA; 9 Division of Pulmonary and Critical Care Medicine, Massachusetts General Hospital, Charlestown, MA USA; 10 Harvard Medical School, Boston, MA USA

**Keywords:** Gene delivery, Viral vectors

## Abstract

*Ex-vivo* gene therapy using stem cells or T cells transduced by retroviral or lentiviral vectors has shown remarkable efficacy in the treatment of immunodeficiencies and cancer. However, the process is expensive, technically challenging, and not readily scalable to large patient populations, particularly in underdeveloped parts of the world. Direct *in vivo* gene therapy would avoid these issues, and such approaches with adeno-associated virus (AAV) vectors have been shown to be safe and efficacious in clinical trials for diseases affecting differentiated tissues such as the liver and CNS. However, the ability to transduce lymphocytes with AAV *in vivo* after systemic delivery has not been carefully explored. Here, we show that both standard and exosome-associated preparations of AAV8 vectors can effectively transduce a variety of immune cell populations including CD4^+^ T cells, CD8^+^ T cells, B cells, macrophages, and dendritic cells after systemic delivery in mice. We provide direct evidence of T cell transduction through the detection of AAV genomes and transgene mRNA, and show that intracellular and transmembrane proteins can be expressed. These findings establish the feasibility of AAV-mediated *in vivo* gene delivery to immune cells which will facilitate both basic and applied research towards the goal of direct *in vivo* gene immunotherapies.

## Introduction

Gene therapy using *ex-vivo* transduction of patient-derived cells has revolutionized medicine, particularly in the case of readily accessible blood hematopoietic stem cells or lymphocytes to treat immunodeficiencies or cancer^1–3^. However, the laborious workflow and infrastructure required makes these therapies expensive, time-consuming, and unscalable. Moreover, the manipulation of cells outside the body may introduce undesirable phenotypic changes^4^. Administering vectors directly into patients would solve most of these problems, potentially expanding the reach of gene therapies to large patient populations anywhere in the world, but unfortunately, such therapies have not advanced due to the absence of a proven and effective delivery system.

AAV vectors were thoroughly developed over the past 35 years and are now demonstrating remarkable, life-changing efficacy in clinical trials for several diseases, including recently FDA approved treatments for a form of hereditary blindness and spinal muscular atrophy^5–9^. The majority of AAV-mediated gene therapies have focused on differentiated cells such as muscle^10,11^, neurons, astrocytes^12^, and liver^13^. AAV has generally been considered inefficient at transducing T cells; however, a recent study revealed that CD3^+^ T cells are a natural reservoir for multiple wild type AAV serotypes^14^. Here we demonstrate that AAV8 vectors can mediate transgene expression in multiple immune cell types after systemic delivery in mice, providing an important proof of concept for developing these vectors for use in *in vivo* therapies.

## Results

### GFP expression in immune cells after systemic delivery of AAV8 and exo-AAV8 vectors in adult mice

**Figure 1:**
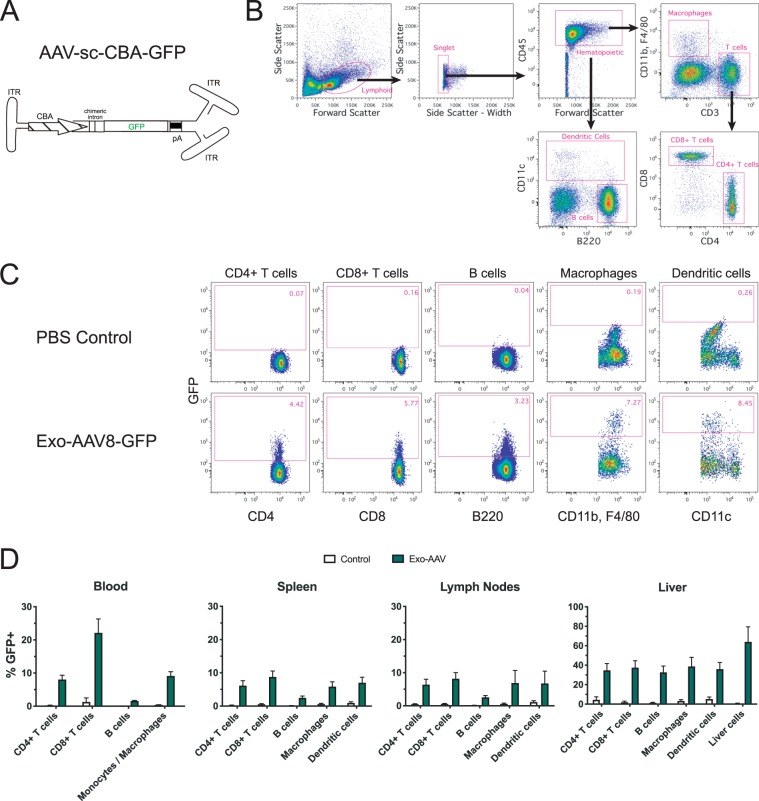
GFP is detected in multiple immune cell types after systemic injection of exo-AAV8-GFP in adult mice. (**A**) Schematic of self-complementary (sc) AAV construct used in this study. CBA, hybrid CMV immediate early, chicken beta actin promoter; GFP, green fluorescent protein; pA, polyadenylation signal; ITR, inverted terminal repeat. Adult male C57BL/6 mice were injected with PBS or 3 × 10^12^ gc (1.2 × 10^14^ gc/kg) exo-AAV8-CBA-GFP and euthanized on day 12 for analysis of tissues. **(B)** Representative gating strategy used to analyze different immune cell populations from the spleen. **(C)** Representative flow cytometry data showing GFP expression in indicated immune cell populations from the spleen at day 12 post-injection. **(D)** Quantitative summary of GFP expression in indicated cell populations in indicated tissues at day 12 post-injection. Data represent mean values ± SEM from n = 6 mice for PBS and n = 7 mice for exo-AAV8-GFP groups collected across three independent experiments.

We previously demonstrated that exosome-associated AAV (exo-AAV) vectors mediate higher transduction efficiencies of target organs compared to conventional AAV vectors when delivered into mice^15–19^. To determine whether these vectors could mediate *in vivo* transduction of immune cells when delivered systemically, we injected C57BL/6 mice intravenously with 3 × 10^12^ genome copies (gc, 1.2 × 10^14^ gc/kg) of an exo-AAV8 vector encoding a self-complementary (sc) green fluorescent protein (GFP) transgene expression cassette under control of the strong, ubiquitous chicken beta actin (CBA) promoter (AAV-sc-CBA-GFP) (Fig. 1A), or PBS as a control. Twelve days post injection, we isolated immune cells from the spleen, lymph nodes, and liver, and assessed GFP expression in CD4^+^ T cells, CD8^+^ T cells, B cells, macrophages, and dendritic cells by flow cytometry (Fig. 1B–D).

GFP expression was detected in all cell types in all tissues studied, demonstrating the ability of exo-AAV vectors to transduce immune cells *in vivo*. In the spleen and lymph nodes, GFP was expressed in 6.2–6.4% of CD4+ T cells, 8.2–8.8% of CD8+ T cells, 2.5–2.6% of B cells, 5.9–6.9% of macrophages, and 6.8–7.0% of dendritic cells. Strikingly, the extent of expression was substantially higher for all 5 cell types in the liver, with percentages of GFP-positive cells ranging from 32.6–38.7% (Fig. 1D). As expected, Non-hematopoietic liver cells (defined as CD45-negative) were also efficiently transduced, with 64.0% of the cells expressing GFP.

**Figure 2:**
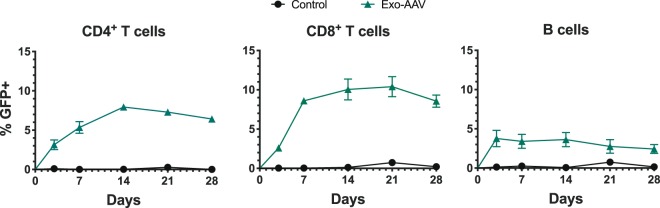
GFP is maintained in circulating blood lymphocytes for several weeks after systemic injection of exosome-associated AAV8-GFP in adult mice. Adult male C57BL/6 mice were injected intravenously with PBS or 9.1 × 10^11^ gc (3.64 × 10^13^gc/kg) of exo-AAV8-CBA-GFP and bled on days 3, 7, 14, 21, and 28. GFP expression in indicated cell populations is shown quantitatively over time. Data represent mean values ± SEM from n = 3 mice for PBS and n = 3 mice for exo-AAV8-GFP groups.

To investigate the kinetics of transgene expression, we injected mice with 9.1 × 10^11^ gc (3.64 × 10^13^gc/kg) of exo-AAV8-sc-CBA-GFP and analyzed immune cells from the blood at days 3, 7, 14, 21, and 28 post-injection. For CD4^+^ and CD8^+^ T cells, the percentage of GFP positive cells peaked at day 14 and slowly declined through 28 days. The peak of transduction efficiency in B cells was day 3 which remained relatively steady through two weeks and then showed a minor decline through 28 days (Fig. 2).

**Figure 3:**
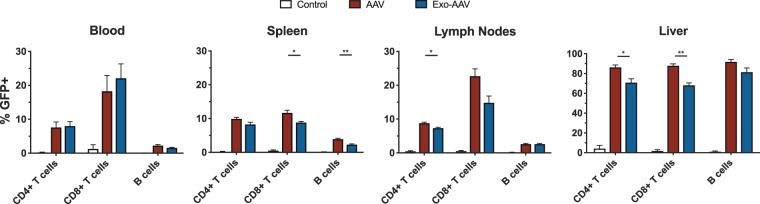
GFP is detected in multiple lymphocyte populations after systemic injection of conventional or exosome-associated AAV8-GFP in adult mice. Adult male C57BL/6 mice were injected intravenously with PBS or 1 × 10^12^ gc (4 × 10^13^ gc/kg) of either standard AAV8-CBA-GFP or exo-AAV8-CBA-GFP and euthanized on day 12. Flow cytometric analysis of GFP expression in indicated cell populations in indicated tissues is summarized. Data represent mean values ± SEM from n = 3 mice for PBS and n = 3 mice for each vector group. *p < 0.05, **p < 0.01 for unpaired t-tests between indicated groups.

To directly compare the transduction efficiency of conventional AAV and exo-AAV vectors, we injected mice with equal doses of these vectors containing the same sc-CBA-GFP construct. As shown in Fig. 3, both vectors mediated similar transduction levels in the selected immune cells, with conventional AAV yielding slightly higher percentages in some cell populations.

**Figure 4:**
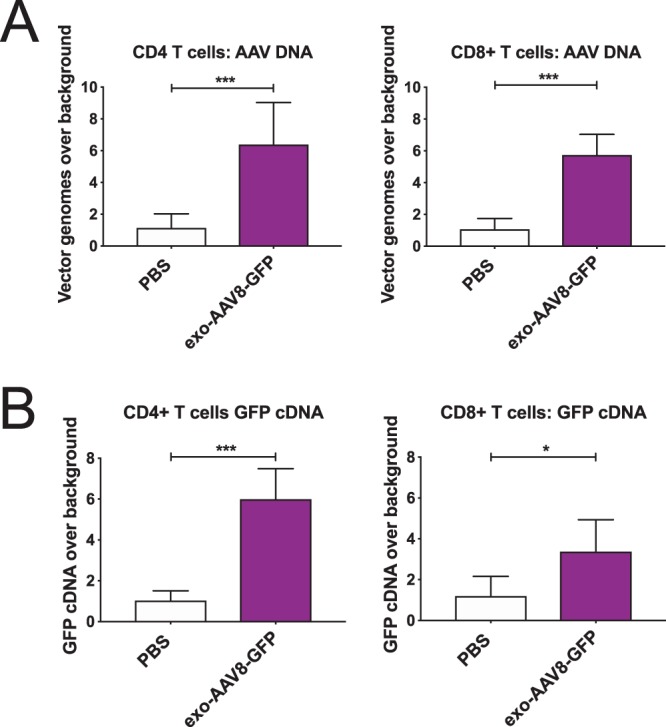
GFP vector transgene and mRNA are detected in T cells after systemic injection of exo-AAV8-CBA-GFP in adult mice. CD4^+^ and CD8^+^ T cells were flow-sorted to detect **(A)** AAV genomes by qPCR and **(B)** vector expressed GFP mRNA by RT-qPCR. All Ct values were normalized to GAPDH values and normalized values for exo-AAV8-GFP injected mice compared to background levels in PBS-injected mice. For (A), average Ct values with GFP probe in PBS-treated mice was cycle 32.99 * for CD8 T cells, cycle 33.49 for CD4 T cells. For (B), average Ct values with GFP probe in PBS-treated mice was cycle 34.36 for CD8 T cells, cycle 34.97 for CD4 T cells. n = 4 for PBS, n = 6 for exo-AAV8-GFP; * p < 0.05, *** p ≤ 0.005.

To provide additional evidence for the direct transduction of immune cells by exo-AAV8, we sorted CD4^+^ and CD8^+^ T cells from the spleens of day 12 mice and isolated DNA to detect the GFP cassette from AAV genomes by qPCR, and RNA to detect GFP mRNA by qRT-PCR. For CD4+ and CD8+ T cells respectively, we found 6.4 and 5.8-fold higher levels of AAV genomes in exo-AAV8-GFP injected mice over PBS-injected controls (i.e. background levels of detection) (Fig. 4A). Similarly, we detected 6.0 and 3.4-fold higher levels, respectively, of GFP cDNA in exo-AAV8-GFP injected mice (Fig. 4B). These findings support our flow cytometry data, and collectively demonstrate that systemically administered exo-AAV8 vectors can mediate detectable transduction of immune cells *in vivo*.

### Expression of human IL-2Rα on the surface of lymphocytes after systemic delivery of exo-AAV8 vector

**Figure 5:**
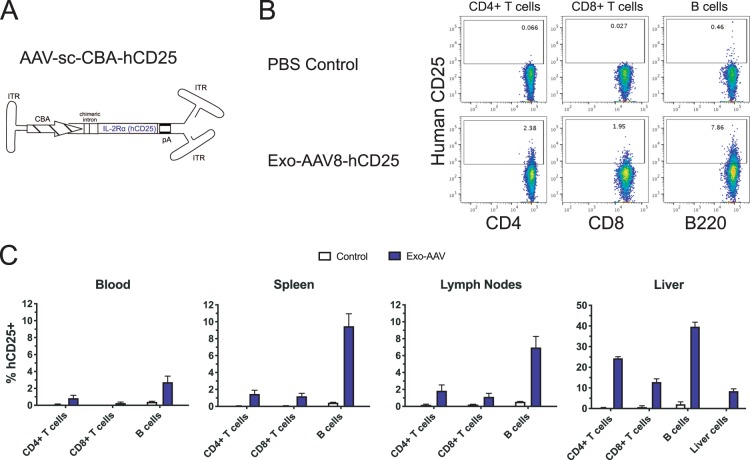
Human IL-2Rα (hCD25) is detected in multiple lymphocyte populations after systemic injection of exo-AAV8-hCD25 in adult mice. (**A**) Schematic of self-complementary (sc) AAV construct used in this study, consisting of the same elements as for Fig. 1A except that the GFP cassette is replaced with hCD25. Mice were injected i.v. with PBS or 1 × 10^12^ gc of exo-AAV8-hCD25 (4 × 10^13^ gc/kg) and 12 days later mice were euthanized for analysis of cells in different tissues. **(B)** Representative flow cytometry data showing hCD25 expression in indicated lymphocyte populations in the spleen using the gating strategy outlined in Fig. 1B. **(C)** Quantitative summary of hCD25 expression in each cell population in each tissue. Data represent mean values ± SEM from n = 3 mice for PBS and n = 3 mice for exo-AAV8-hCD25 groups.

As many immune cell therapies (e.g. CAR T cells) modify or express ligands on the surface of lymphocytes, we tested the feasibility of expressing a human receptor on the surface of mouse immune cells using AAV-mediated *in vivo* transduction. We constructed a sc AAV plasmid encoding human interleukin-2 receptor alpha chain (IL-2Rα), also known as CD25, and produced exo-AAV8-sc-CBA-hCD25 vector (Fig. 5A). We injected mice intravenously with 1 × 10^12^ gc (4 × 10^13^ gc/kg) of exo-AAV8-sc-CBA-hCD25 or PBS as a control. At day 12 post-injection, CD4^+^ T cells, CD8^+^ T cells, and B cells from the blood, spleen, lymph nodes, and liver were analyzed for hCD25 (hCD25) expression by flow cytometry (Fig. 5B,C).

We found detectable expression of hCD25 on all three lymphocyte populations, with 0.8–1.9% of CD4^+^ T cells, 0.3–1.2% of CD8+ T cells, and 2.7–9.5% of B cells in the blood, spleen, and lymph nodes expressing hCD25. Again, these levels were substantially higher in the liver, with 24.4% of CD4 T cells, 12.9% of CD8+ T cells, and 39.7% of B cells expressing hCD25. Interestingly, the transduction of liver cells was much lower, with only 8.4% of these cells expressing hCD25. These results demonstrate the ability of AAV vectors to mediate *in vivo* transduction and expression of a surface receptor on immune cells.

## Discussion

In this study, we explored the use of AAV vectors to mediate expression of transgene products in lymphocytes after systemic delivery in mice. We observed readily detectable expression of two genes of interest (GFP, and a cell surface receptor) out to at least 28 days post injection.

We initiated our study with exosome-associated AAV (exo-AAV) vectors as we have previously demonstrated higher levels of transduction of target organs compared to standard AAV vectors^15–21^. After our initial observation that we could transduce lymphocytes with systemically injected exo-AAV8-GFP we performed a dose-matched study with conventional AAV8. Surprisingly, similar levels of transduction were observed between exo-AAV8 and AAV8, with conventional AAV8 transducing slightly more cells. In this study we used self-complementary (sc) vectors as well as relatively high doses of AAV (4 × 10^13^ gc/kg), both factors which may have minimized any boost in transduction observed with exo-AAV. For example, our earlier study demonstrating superior hepatocyte transduction by exo-AAV8^15^ was achieved at much lower doses (~4 × 10^10^ gc/kg vs. 4 × 10^13^ gc/kg) and also with a single stranded AAV genome. This suggests that the enhanced transduction efficiency of exo-AAV may be limited to certain cell types or lower doses of vector.

Since we observed transduction of lymphocytes using either conventional AAV8 and exo-AAV8 vectors, both types could be pursued for clinical development. Since conventional AAV8 vectors are already in clinical trials, there are currently less regulatory and manufacturing hurdles to overcome than for exo-AAV vectors. Systemically injected AAV8 vectors are currently used in clinical trials for hemophilia B^22^, so the same clinical protocols and limitations should apply for the transduction of lymphocytes using AAV8. Some of these protocols use short-term, high-dose corticosteroid treatments to limit development of anti-AAV8 CTL responses. However, patients with pre-existing anti-AAV8 neutralizing antibodies are often excluded. The use of exo-AAV vectors, which can shield AAV capsids from host antibodies, may provide an attractive alternative option.

While the doses in the current study are high (up to 1.2 × 10^14^ gc/kg tested), these are clinically-relevant doses used for systemically delivered AAV vectors^8^. Importantly, all treated mice (total of 19 exo-AAV/AAV-injected animals in these experiments) survived the duration of the study with no signs of toxicity. Future preclinical toxicology studies will further validate the safety of this approach. Currently, the high dose required to achieve appreciable transduction of lymphocytes by the exo-AAV8/AAV8 vectors is a weakness of the system. At these doses, the ubiquitous CBA promoter in the transgene cassette results in high levels of collateral expression in peripheral organs (heart, liver, muscle). However, this can be ameliorated in the future at two levels. The first is by directly targeting the exo-AAV vector to lymphocyte ligands or selecting new AAV capsids with improved transduction efficiencies for these cells, enabling us to use much lower doses. The second is by transcriptional/post-transcriptional targeting/detargeting transduced cells. This can be done by using tissue-specific promoters that express well in lymphocytes but not in peripheral organs. Additionally, detargeted expression by vectors can be achieved by placing microRNA (miR) recognition sites in the AAV vector of miRs highly expressed in organs such as liver, but not the target cells^23^.

Another surprising finding of our study was the higher percentages of GFP expressing liver resident lymphocytes compared to spleen or lymph nodes. This raises the possibility that accumulation of vector in the liver may contribute to higher transduction efficiencies in resident immune cell populations. Alternatively, some GFP protein/mRNA may be transferred from highly-transduced hepatocytes to local lymphocytes^24^. Future studies employing AAV transgene cassettes which allow detargeting from hepatocytes with miR seed sequences^25^ or T cell specific promoters^26^, may help delineate this phenomenon.

We assessed the kinetics of transgene expression by performing serial bleeds of mice and assessing lymphocytes for GFP expression throughout a monthlong timecourse. While expression in CD4^+^ T cells and CD8^+^ T cells peaked at day 14 and declined out to day 28, it peaked at day 3 in B cells with a slower decline over time. This may reflect cell-type differences in vector uptake and/or kinetics of transgene expression. The decline over time suggests that expression is largely mediated by episomally-maintained AAV that is diluted out over time upon cell division rather than the small percentage of AAV that stably integrates into the genome *in vivo* (~0.1%)^27–29^.

We observed an apparent discrepancy between the transduction profiles between the cytoplasmic reporter gene GFP and the transmembrane protein hCD25. CD8+ T cells exhibited the lowest percentages of hCD25 expression and B cells the highest, which was reversed from our findings with GFP. Moreover, a lower percentage of liver cells exhibited surface expression of hCD25 than T and B cells, in contrast to the experiment with GFP transgene, in which liver cells had higher percentages of GFP compared to T and B cells. The discrepancy in transgene expression may be due to differences between these cell types in their ability to process and translocate transmembrane receptors to the cell surface, or differential effects of the transgenes on the survival and/or proliferation of these cell types. Alternatively, since hCD25 is a transmembrane protein, it is likely present on the surface of the exo-AAV which may have altered the target cell tropism of the particle compared to exo-AAV-GFP without surface modification.

Collectively, our findings demonstrate that AAV vectors can mediate transduction and expression of both intracellular and transmembrane proteins in lymphocytes following systemic administration in mice. These results establish early feasibility of direct *in vivo* gene modification of immune cells. In support of this, a very recent publication utilizing a very sensitive Cre recombinase-based reporter system demonstrated that a systemically injected AAV8 vector could transduce lymphocytes in the spleens of immune competent mice, albeit at efficiencies much lower than what we obtained^30^. Moreover, Münch *et al*. expressed a CD4-binding DARPin molecule on the AAV capsid to specifically target human CD4+ T cells in a humanized mouse model, and showed that very low doses (5 × 10^9^ gc/mouse) could deliver a luciferase transgene that generated a bioluminescence signal localized precisely to the spleen^31^. Finally, the Zimmermann group has shown that intrathymic (i.t.) injection of AAV vectors into mice can achieve up to 5% thymocyte transduction efficiency^32^, and this can be used to correct immunodeficiency in *ZAP70*-deficient mice.

Lentivirus vectors have demonstrated remarkable efficacy in the transduction of peripheral blood mononuclear cells (PBMC) *ex-vivo* for the generation of CAR T cells^33^, and more recently, two studies from the Buchholz group demonstrated their ability to mediate direct *in vivo* modification of CD4^+^ and CD8^+^ T cells in humanized mouse models when used in conjunction with cell targeting strategies^34,35^. Transduction levels were in the range of 0.5–5%, similar to the transduction efficiencies we obtained with exo-AAV in the spleen, lymph nodes, and blood. Notably, in both lentivirus studies, efficient transduction required activation of the PBMCs. Jamali *et al*. used a transduction-enhancing cationic peptide to further increase the efficiency of the targeted lentivirus system in cultured cells^36^.

Both the lentivirus and AAV-based vectors have unique advantages and caveats for *in vivo* gene delivery. One obvious advantage of lentivirus vectors over AAV is the packaging capacity (~9 kb for lentivirus and 4.7 and 2.3 kb for single-stranded and self-complementary AAV genomes, respectively). Also, lentivirus integrates into the chromosome and in general, will mediate stable transduction of cells. In cases where long-term expression is desired, this is an important positive feature. In contrast, AAV vector genomes mainly persist episomally as concatamers^37^, so AAV genomes will be diluted in expanding T and B cell populations, resulting in waning transgene expression over time^38^. It is possible, however, that over time, low-frequency integration events lead to stable lymphocyte clones. We expect this to be an infrequent event; however future preclinical studies performed over longer intervals can assay for an increase in percentage of transduced cells at later timepoints and look at whether these cells are clonal and contain integration of the full transgene cassette necessary for expression. In applications such as CRISPR/Cas9 editing, short-term expression is desired to reduce potential off-target editing. Moreover, many T cell engineering strategies (e.g. CAR T cells) may also benefit from transient expression to limit adverse events from unregulated immune responses. From a practical standpoint, AAV vectors have already established an extensive safety profile for *in vivo* gene therapy in humans compared to lentivirus vectors, which have mostly been used for *ex-vivo* gene therapy thus far.

Our validation of exo-AAV vectors for *in vivo* delivery introduces additional potentialities, including reduced host antibody recognition of AAV particles^15,18^, and the prospect of cell targeting strategies through decoration of exosomes with relevant ligands^18^, similar to the work done with targeted lentivirus vectors^39^.

## Methods

All methods and experiments were approved by the Partners Institutional Biosafety Committee of Partners Healthcare (Massachusetts General Hospital).

### AAV constructs

AAV transgene plasmid (AAV2 inverted terminal repeat (ITR)-flanked) encoding green fluorescent protein (GFP) under the hybrid CMV immediate-early/chicken beta actin (CBA) promoter, AAV-CBA-GFP, was kindly provided by Dr. Miguel Sena-Esteves (UMass Medical Center). AAV-CBA-GFP encodes a self-complementary (sc) AAV genome.

### exo-AAV and AAV preparations

exo-AAV were produced in 293 T cells as previously described^21^. Briefly, a triple transfection of AAV plasmid, rep/cap plasmid (AAV8 serotype used) and helper plasmids (pAdΔF6) was performed using the calcium phosphate method in ten 15 cm dishes. Media was changed to 2% FBS in DMEM the day after transfection and to exosome-free 2% FBS at 48 h post transfection. At 72 h post transfection, media was harvested. Cell debris and apoptotic bodies were removed by sequential, 10 min 300 x g and 2000 x g centrifugations, respectively. The supernatant containing exo-AAV was then centrifuged at 20,000 x g for 1 h to deplete larger microvesicles. Next, the remaining media was centrifuged at 100,000 x g for 1 hour using a Type 70 Ti rotor in an Optima™ L-90K ultracentrifuge (both Beckman Coulter, Inc., Indianapolis IN). The resulting pelleted material was resuspended in serum-free, antibiotic-free DMEM. Conventional AAVs were purified from the cell lysate using iodixanol-gradient ultracentrifugation. Vectors were stored at −80 °C until use. Before titration, exo-AAV sample was treated with DNase to remove plasmid DNA from the transfection by mixing 5 µl of the sample with 1 µl DNase I, 5 µl 10x buffer, and 39 µl water. Samples were incubated 1 h at 37 °C and then Dnase I was inactivated at 75 °C for 15 min. We purified AAV genomes using High Pure Viral Nucleic Acid Kit (Roche, Indianapolis, IN). Next, exo-AAV preparations were titered using a quantitative TaqMan PCR that detects AAV genomes (polyA region of the transgene cassette) as previously described^40^.

### Mice

C57BL/6J mice were purchased from the Jackson Laboratory (Bar Harbor, ME). Blood, spleens, lymph nodes, and livers were harvested from 8–12 week old male and female mice. All animal experiments were approved by the Institutional Animal Care and Use Committee of Massachusetts General Hospital. We confirm that all experiments were performed in accordance with relevant guidelines and regulations.

### Flow cytometry

Blood samples were collected in microfuge tubes containing EDTA and subjected to red cell lysis with ACK buffer (Corning). Single cell suspensions were prepared from spleen, lymph node, and liver samples by mechanical disruption. All samples were stained with antibodies at 4 °C for 30 min. Flow cytometry was performed on the LSRII, Fortessa (both BD Immunocytometry Systems), or Cytoflex S (Beckman Coulter). Cell sorting was performed on the FACSAria II (BD Immunocytometry Systems). Flow data was analyzed with FlowJo software (Treestar). All antibodies were purchased from BioLegend or Life Technologies.

### RNA isolation and GFP cDNA synthesis

Total RNA was isolated from 10^4^ sorted GFP^+^ T cells using the RNeasy® Plus Micro Kit (Qiagen) according to manufacturer’s instructions. RNA was eluted in nuclease-free water and stored at −80 °C until use. cDNA was synthesized using 3 µl of RNA with the SuperScript® VILO™ cDNA synthesis kit (Invitrogen) according to manufacturer’s protocol.

### DNA isolation

Total genomic DNA and viral DNA were purified from approximately 10^5^ sorted T cells using the DNeasy® Blood and Tissue Kit (Qiagen) according to manufacturer’s instructions. DNA was stored at −20 °C until use.

### GFP qPCR

To detect GFP cDNA in T cells and liver samples we used TaqMan® Gene Expression Assays with two probe and primer sets, one for enhanced GFP (Mr04329676_mr Enhance) and mouse GAPDH (Mm99999915_g1) (ThermoFisher). We used 1 ul of the cDNA reaction in a qPCR mastermix using TaqMan™ Fast Universal PCR Master Mix (2x), no AmperErase™ UNG (ThermoFisher). Each cDNA sample was run separately in both a GAPDH and GFP Taqman probe and primer reaction in an ABI7500 Fast Thermalcycler.

### Statistics

Statistical analysis was performed using GraphPad Prism (v6.01; LaJolla, CA). A p-value ≤ 0.05 was considered statistically significant. For analysis between two groups, an unpaired t test was utilized.

## Data Availability

Raw data that support the findings of this study are available from the corresponding authors. Plasmid constructs described in this publication are available upon completion of a standard Material Transfer Agreement with The Massachusetts General Hospital.
